# Efficacy of microwave ablation for severe secondary hyperparathyroidism in subjects undergoing hemodialysis

**DOI:** 10.1080/0886022X.2016.1256307

**Published:** 2016-11-15

**Authors:** Zongli Diao, Liyan Wang, Dishan Li, Wenhu Liu

**Affiliations:** Department of Nephrology, Beijing Friendship Hospital, Capital Medical University, Beijing, China

**Keywords:** Microwave ablation, secondary hyperparathyroidism, hemodialysis, efficacy, ultrasonography

## Abstract

Severe secondary hyperparathyroidism (SHPT) is a serious problem in patients undergoing hemodialysis. The efficacy and safety of microwave ablation (MWA), a minimally invasive treatment, for severe SHPT are as yet unclear. To clarify the role of MWA, we administered it to patients with severe SHPT and assessed its efficacy and safety. This was a prospective, single-center, single-arm, clinical trial. We enrolled patients with severe SHPT attending our hemodialysis center who met the inclusion and exclusion criteria. We then assessed primary outcome measures (serum concentrations of intact parathyroid hormone) and secondary outcome measures (serum concentrations of calcium and phosphorus). Twenty-six patients were enrolled in this study, 10 of whom (38.46%) were responsive to MWA and 16 (61.54%) of whom were not. The main complication was hypocalcemia (10 cases, 38.46%), which had occurred in all cases by one week after administration of MWA. Responding patients with hypocalcemia all achieved normal serum calcium concentrations within seven months and non-responding patients within three months. There were no changes in serum phosphorus concentrations after MWA in either responders or non-responders. Microwave ablation is relatively ineffective in patients with severe SHPT undergoing maintaining hemodialysis and should not be the initial therapy in such cases.

## Introduction

Secondary hyperparathyroidism (SHPT) is a common and serious problem in patients undergoing maintenance hemodialysis. The major associated pathophysiological changes are high serum concentrations of intact parathyroid hormone (iPTH), and disorders of calcium (Ca) and phosphorus (P) metabolism; these are associated with increased mortality.^1–3^

Parathyroidectomy is the standard treatment for severe SHPT.^4^^,^^5^ Microwave ablation (MWA), which has become an increasingly popular minimally invasive means of treating tumors,^6^ has also been used to treat severe SHPT, as was first reported in 2013.^7^ In that study, MWA was used to treat SHPT with parathyroid nodules larger than 1.5 cm. At the 12-month follow-up, the mean iPTH concentration had decreased from 3200 pg/mL to 1000 pg/mL. However, these researchers reported only the outcomes of combined MWA and radiofrequency ablation, not outcomes for each modality separately. In another retrospective study, iPTH concentrations decreased from a pretreatment mean of 1570 pg/mL (651–6800 pg/mL) to 419 pg/mL (21–1500 pg/mL) at the 12-month follow-up.^8^ Although the mean iPTH post-treatment concentration was in the target range (about 150–600 pg/mL),^9^ the range was from 21 to 1500 pg/mL, indicating that some participants were non-responsive. Furthermore, neither of these studies reported efficacy in terms of the percentage of participants who were responders and details of relevant outcomes. The goal of this prospective study was to determine the efficacy and safety of MWA in patients with severe SHPT undergoing maintenance hemodialysis.

## Methods

### Study design

This prospective, single-center, single-arm, open-label clinical trial was designed to test the efficacy and safety of MWA in patients with severe SHPT undergoing maintenance hemodialysis. The study was approved by the institutional review board of Beijing Friendship Hospital, Capital Medical University (No. 20120179) and written informed consent was obtained from all study patients. This study was registered with ClinicalTrials.gov (No. NCT01640184).

### Study subjects

Patients undergoing maintenance hemodialysis between 1 January 2013 and 31 December 2014 in our hospital were eligible to participate. Specific inclusion and exclusion criteria were as follows.

#### Inclusion criteria

(1) Age: 18–75 years. (2) Had been undergoing maintenance hemodialysis ≥6 months. (3) Severe SHPT: high serum iPTH concentrations (>800 pg/mL) and resistance to combination therapy with a phosphorus-restricted diet, phosphate binder, and calcitriol.

#### Exclusion criteria

(1) Previous parathyroidectomy. (2) Known history of parathyroid or other neoplasms in the neck region. (3) Treatment with cinacalcet within the previous three months. (4) Currently participating in other clinical trials.

### Intervention

All participants consented to receiving MWA. A single ultrasound specialist administered the MWA therapy to all subjects using a KY2000 ablation system (Nanjing Kangyou Applied Research Institute, Nanjing, China) at a frequency of 2450 MHz; the ultrasound was guided in all subjects by another single ultrasound specialist.

Briefly, the procedure was as follows. First, a parathyroid ultrasound was performed to determine the blood supply of the parathyroid nodule(s) and the ablation path. Second, after the neck skin had been sterilized, local anesthesia was induced with 2% lidocaine hydrochloride, following which 5 mL 2% lidocaine hydrochloride was diluted to 20 mL and then injected into the area around the parathyroid nodule(s) to create a heat insulation layer to protect the adjacent nerves and blood vessels. Third, an ablation needle was inserted into the parathyroid tissue under ultrasound guidance. Ablation was then performed, the MWA power being between 25 and 35 W according to target nodule size. When hypoechoic signals were obtained from the whole nodule(s) and no flow signals were detected, ablation was terminated.

### Outcomes

Serum iPTH, Ca, and P concentrations were measured at baseline (within one month pre-ablation), immediately (within 1 h after MWA), one week (±3 days), and one month (±3 days) after ablation, and then bimonthly.

#### Primary outcome measures

Outcomes were classified as Response or No Response.

The target value of 124–558 pg/mL for iPTH concentration was chosen according to Kidney Disease: Improving Global Outcomes (KDIGO) guidelines.^10^ Response was defined as serum iPTH concentration in the target range for six consecutive months after MWA, with or without calcitriol therapy, and No Response as serum iPTH concentration greater than 558 pg/mL after MWA, and resistant to calcitriol for three consecutive months.

#### Secondary outcome measures

Secondary outcome measures were the target serum Ca (2.0–2.5 mmol/L) and P concentrations (0.97–1.62 mmol/L) in the KDIGO guidelines^9^: The targets for serum Ca and P concentrations were defined as achieving concentrations in the target range on more than 75% of follow-up checks.

Follow-up was ended when either death or recurrence occurred.

### Statistical analysis

All statistical analyses were performed with SPSS 19.0 (SPSS Inc., Chicago, IL). All data with normal distribution are expressed as the mean (±standard deviation), and all data with skewed distribution as the median (interquartile range). To compare changes in serum iPTH, Ca, and P concentrations before and after MWA, analysis of variance of repeated measures was used when the data were normally distributed and Wilcoxon tests when not normally distributed.

## Results

### Study subjects and intervention

Of the 351 patients receiving hemodialysis during 2013–2014 in our hemodialysis center, 27 (7.7%) were enrolled in this study, one of whom was excluded for refusing to undergo lab tests. Thus, data for 26 subjects (male:female, 10:16) of average age 55.1 (±9.6) years and duration of dialysis 87.0 (±46.0) months were assessed.

All parathyroid gland nodules were ablated in patients with one, two, or three nodules. All nodules were ablated in two of the four patients with four parathyroid nodules and two in each of the other two patients, the remaining two in each patient being located too deeply to permit ablation. The number of ablated nodules averaged two per patient. The power of MWA administered was 32.9 ± 5.13 W.

### Primary and secondary outcomes

**Figure 1.**
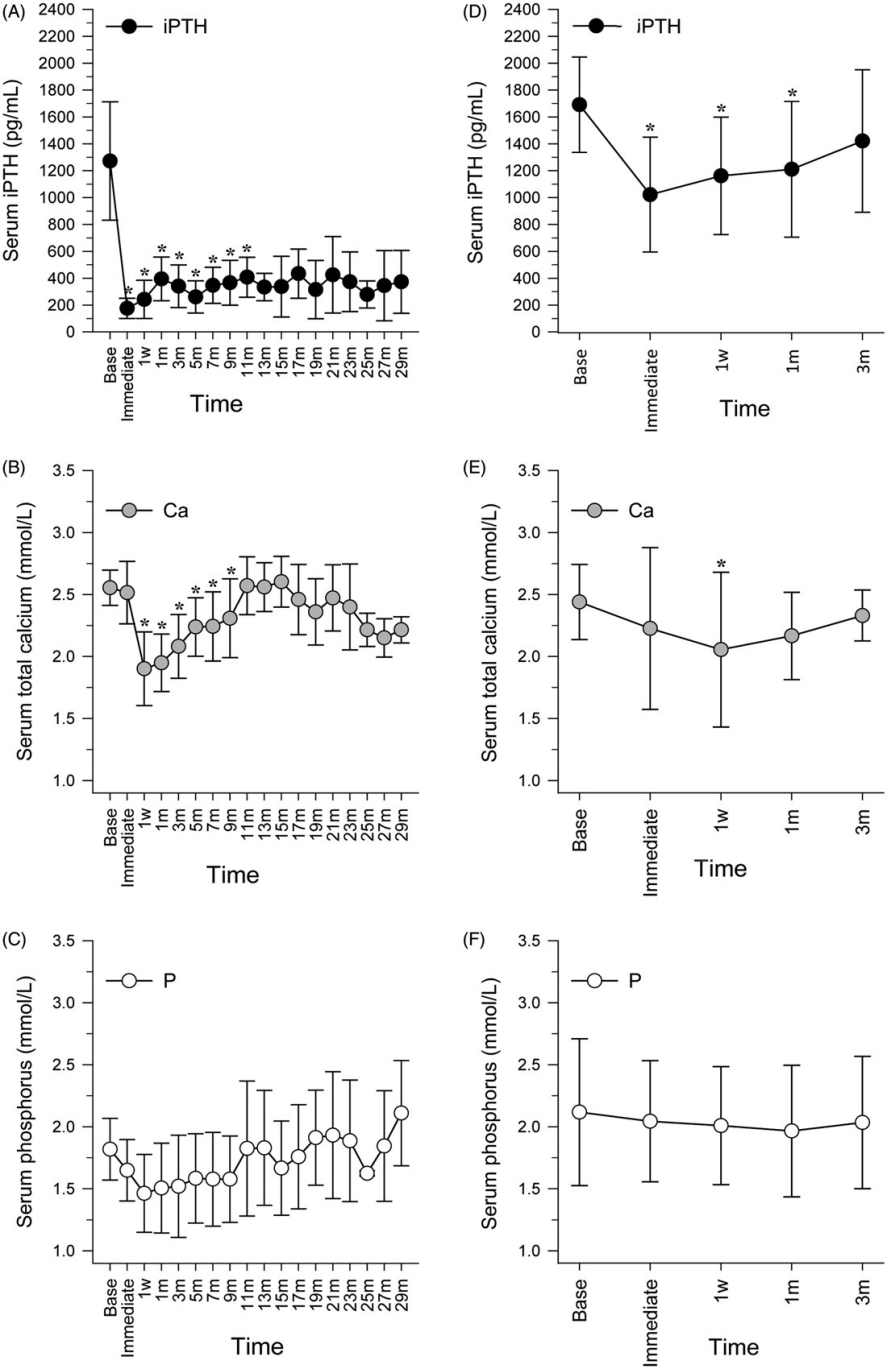
Changes in serum concentrations of iPTH, Ca, and P after MWA in responders (A, B, and C) and non-responders (D, E, and F). As fewer than six responders were followed up for more than 13 months, findings for responders are only shown up to 13 months. Follow-up ended at three months for non-responders, when they were identified as non-responders. *Statistically significant difference compared with baseline values (*p*<.05). Immediate: immediately after MWA; w: week; m: month.

The primary and secondary outcomes are displayed in Table 1 and Figure 1. Ten patients who achieved Response were followed up for 16 (7–24.5) months. In these patients, serum iPTH concentrations declined to within the KDIGO target range (124–558 pg/mL) immediately after MWA, the changes compared with baseline values being statistically significant (1272.02 ± 440.34 vs. 176.09 ± 75.84 pg/mL, *p*<.001, Figure 1(A)). During follow-up, the iPTH concentrations of seven of these patients remained within the KDIGO target range; however, the remaining three patients experienced recurrences at the 15, 19, and 21-month follow-up. Compared with baseline values, their total serum calcium concentrations declined significantly at one week after MWA (2.56 ± 0.14 vs. 1.90 ± 0.30 mmol/L), this difference being statistically significant (*p*<.001) (Figure 1(B)). From one week to nine months after MWA treatment, all calcium concentrations were significantly lower than those at baseline; however, they started to increase from the one-week follow-up and did not differ significantly from baseline values by the 11-month follow-up. There were no significant differences between baseline and follow-up serum phosphorus concentrations (Figure 1(C)).

**Table 1.** Rates of achieving target outcomes after MWA in patients with severe SHPT undergoing hemodialysis.

		Before MWA	After MWA
Primary outcomes			
	Response	NA	38.46% (10/26)
	Recurrence	NA	30% (3/10)
	No Response	NA	61.54% (16/26)
Secondary outcomes			
Calcium		50% (13/26)	30.77% (8/26)
	Phosphorus	15.38% (4/26)	30.77% (8/26)

NA: not applicable.

Sixteen patients with No Response stopped follow-up at three months (when they were identified as having No Response). Compared with baseline values (1691.42 ± 354.48 pg/mL), iPTH concentrations declined significantly immediately after MWA (1022.02 ±  427.05 pg/mL), and at the one-week (1161.82 ± 437.21 pg/mL) and one-month (1210.61 ± 505.34 pg/mL) follow-ups, *p* values being <.001, .005, and .013, respectively. However, iPTH concentrations did not decline to the target range (Figure 1(D)). Furthermore, they started to increase from immediately after MWA, increasing by the three-month follow-up to concentrations that did not differ significantly from those at baseline. Compared with baseline values, the total serum calcium concentration at the one-week follow-up declined significantly (2.44 ± 0.30 vs. 2.05 ± 0.62 mmol/L, *p*=.021), but increased by the one-month follow-up to concentrations that did not differ significantly from baseline values (2.44 ± 0.30 vs. 2.16 ± 0.35 mmol/L, *p*=.099) (Figure 1(E)). Serum phosphorus concentrations did not change significantly after MWA (Figure 1(F)). One patient accepted three MWAs within 10 months, but the iPTH level immediately after the third ablation was 1064.5 pg/mL. We decided that the patient needed parathyroidectomy, but the patient refused. The other patient accepted a second ablation but the iPTH level immediately after the second ablation was 837 pg/mL. We recommended parathyroidectomy to this patient, and he underwent this procedure one year later. The iPTH level was very low (30.46 ± 8.42 pg/mL) during one-year follow-up.

### Adverse events

The most common complication was hypocalcemia (13 cases, 50%); this was identified at the one-week follow-up. Seven patients with Response (7/10, 70%) and six with No Response (6/16, 37.5%) presented with Ca concentrations of 1.76 (1.58–1.96) mmol/L and 1.86 (1.78–1.93) mmol/L, respectively. These patients were injected with calcium gluconate after each hemodialysis session and/or prescribed calcium carbonate and calcitriol. The calcium concentrations increased to within the normal range at the seven-month follow-up in patients with Response and at three months in those with No Response.

Two patients (one each with Response and No Response) presented with hoarseness after MWA; this resolved spontaneously at two weeks and three months, respectively.

## Discussion

In this study, we assessed the efficacy and safety of MWA in patients with SHPT undergoing maintenance hemodialysis and found that 10/26 patients were responsive and 16/26 non-responsive to it, indicating poor efficacy.

MWA has been widely used in interventional therapy, particularly for treating tumors. Radiofrequency ablation has proven efficacy in treating SHPT.^11^ Like radiofrequency ablation, MWA is a minimally invasive treatment. As it has many other advantages, including a more predictable ablation zone, ability to simultaneously treat multiple lesions, large ablation volumes, and fast ablation times,^10^^,^^12^^,^^13^ we believed it might achieve good results in subjects with SHPT. However, as reported in the Results section, our results were not as good as expected. Possible explanations for this include the following.

First and most importantly, there may have been some residual parathyroid cells after ablation. As this type of ablation is performed under ultrasound guidance and not direct vision, nodules may be incompletely ablated. As has been well established, disorders of calcium and phosphorus metabolism are the most important reasons for patients undergoing hemodialysis developing SHPT, a syndrome that is common in these patients.^14–16^ Thus, with the inevitably ongoing disordered calcium and phosphorus metabolism, any residual parathyroid cells would proliferate and SHPT would recur.

Second, we were unable to ablate all parathyroid nodules in all patients. In two of our patients, each of whom had four nodules, we elected not to ablate two of the four nodules because they were too deep and too close to the carotid artery to ablate them without injuring it. Another consideration is that ectopic parathyroid tissue is present in about 14% of subjects with SHPT^17^; failure to detect some ectopic parathyroid nodules by ultrasound could have led to incomplete ablation.

Third, efficacy is highly dependent on the technique of the operator. The parathyroid glands are close to the recurrent laryngeal nerve, which is not visualized by ultrasound. Operators who believe that a nodule is very close to this nerve may thus reduce the volume ablated to avoid injuring it, resulting in incomplete ablation and lack of response or early recurrence.

This study had several other limitations. First, it was a single-center study and the sample was therefore small. Larger studies are required to clarify the efficacy of MWA in subjects with SHPT. Second, because the ablations were all performed by a single operator, our findings may not be reproducible.

In conclusion, we do not recommend MWA as first-line treatment in patients with severe SHPT undergoing maintenance hemodialysis because it is not sufficiently efficacious. However, further studies are required to more accurately determine the efficacy and safety of MWA in such patients.
